# Ion channels: their discovery, their function, and their role in disease

**DOI:** 10.1186/1471-2164-15-S2-O1

**Published:** 2014-04-02

**Authors:** Erwin Neher

**Affiliations:** 1 Max Planck Institute for Biophysical Chemistry, 37077 Goettingen, Germany

## 

The neurons of our brain are prime targets for drugs and therapeutic interference. More specifically, receptors on the surface of these cells as well as ion channels, which mediate ion flow across membranes, are key regulators of cellular function. Therefore, a small number of drug molecules, acting on such molecules, can have dramatic effects on cells and tissue, as a whole.

The study of transport-related molecules and of their mechanisms of action has received an enormous boost after Bert Sakmann and I developed the 'patch clamp technique' for recording of the ion currents flowing through individual channels. Furthermore, it became obvious that a large number of congenital diseases can be traced back to mutations in ion channels. Studying the effects of such mutations on channel function and relating these changes to clinical manifestations offers ample opportunities to study 'human biology'.

In this lecture I will sketch the early work, which led to the discovery of ion channels in cellular membranes. I will describe the potential of the patch-clamp technique for the study of cellular signaling, and review recent work (by others) on ion channels as molecular targets in drug discovery and disease.

